# VSIG2 hinders gastric cancer progression by suppressing ANXA2-mediated NF-κB pathway activation: VSIG2 hinders gastric cancer progression

**DOI:** 10.3724/abbs.2025202

**Published:** 2025-11-04

**Authors:** Qingfeng Ni, Yang Wang, Xinyue Bian, Qiuchan Qu, Boyuan Shen, Yuanjie Niu, Jiawei Yu, Jianwei Zhu

**Affiliations:** 1 Department of Gastrointestinal Surgery Affiliated Hospital of Nantong University Nantong 226001 China; 2 Department of Women’s Health Care Affiliated Maternity and Child Health Care Hospital of Nantong University Nantong 226001 China

**Keywords:** gastric cancer, VSIG2, K63 polyubiquitination

## Abstract

As the fifth most common cancer and the third leading cause of cancer death worldwide, gastric cancer (GC) has long been a serious global health challenge. The purpose of this study was to explore the expression of V-set and immunoglobulin domain containing 2 (VSIG2) in GC and to elucidate its role in GC progression and related mechanisms. Western blot analysis, qRT-PCR and immunohistochemical (IHC) staining are used to detect the expression of VSIG2 in GC cells and tissues. Kaplan-Meier survival curve analysis is performed. The effects of VSIG2 on biological effects related to GC progression *in vitro* are detected by CCK-8, EdU, Transwell and wound healing assays and *in vivo* by a nude mouse subcutaneous tumor model and a liver metastasis model. Mechanistically, co-immunoprecipitation, immunofluorescence and ubiquitination experiments are used to explore the regulatory effect of VSIG2 on ANXA2 and the regulatory effect between FBXW10 and ANXA2. VSIG2 is abnormally expressed at low levels in patients with GC and is associated with patient prognosis. Low VSIG2 expression is closely related to tumor size, lymph node metastasis, TNM stage and vascular invasion in GC patients. Functionally, *in vitro* and *in vivo* experiments reveal that VSIG2 could inhibit the growth, proliferation and metastasis of GC. Mechanistically, VSIG2 and ANXA2 interact directly in GCs and co-localize at the cell membrane. Further exploration reveals that highly expressed VSIG2 competes with FBXW10 for binding to ANXA2 and relies on FBXW10-mediated K63 polyubiquitination of ANXA2 to induce membrane localization of ANXA2 and further inactivate NF-κB, thereby suppressing GC progression. In summary, VSIG2 is expressed at abnormally low levels in patients with GC, and its low expression is associated with poor patient prognosis. VSIG2 can inhibit the proliferation and migration of GC via the ANXA2/NF-κB pathway. This study elucidates a new mechanism by which VSIG2 inhibits GC progression, which may provide a new perspective for the diagnosis and treatment of GC patients.

## Introduction

Gastric cancer is the fifth most common malignant tumor in the world and the third leading cause of cancer-related death. In 2020, there were 1.09 million new cases and 769,000 deaths worldwide, of which more than 60% occurred in East Asia [1]. *Helicobacter pylori* ( *H*. *pylori*) chronic infection accounts for approximately 78% of all cases and is classified by the WHO as a class I carcinogenic factor. It is expected that 11.9 million gastric cancer cases will be attributed to *H*. *pylori* infection in the cohort born from 2008 to 2017 [2]. Although endoscopic screening, radical surgery and perioperative FLOT regimens have significantly improved the prognosis of patients with resectable disease, the 5-year survival rate of patients with advanced or metastatic gastric cancer is still less than 20% [3]. Molecular mapping studies (TCGA & ACRG) have revealed four subtypes, namely, EBV-positive, microsatellite instability, chromosome instability, and genomic stability, providing a precise framework for the targeting and immunotherapy of HER2, claudin 18.2, VEGFR2, and PD-1/PD-L1. However, tumor microenvironment-mediated immune escape and targeted drug resistance are still the biggest challenges [4]. Therefore, in-depth analysis of the molecular network of gastric cancer development and the development of new joint strategies are urgently needed to improve the survival rate of patients.

V-set and immunoglobulin domain-containing 2 (VSIG2) is an emerging B7-like immune checkpoint whose expression topography has quietly shifted from a “thymocyte relic” to a decisive regulator of tumor–immune crosstalk [5]. First cloned as the Xenopus cortical thymocyte marker CTXL, human *VSIG2* maps to 11q24.2 and encodes a 283-aa type-I glycoprotein that pairs a membrane-distal IgV with a juxtamembrane IgC2 domain [6]. Transcriptomic atlases now reveal that VSIG2 is not confined to the thymus but is highly enriched in gastric and colonic epithelia, renal tubules, and critically tumour-associated macrophages and B cells [7]. Functionally, VSIG2 behaves as a Janus-faced rheostat: in pancreatic ductal adenocarcinoma, VSIG2 partners with LAMTOR2 to hyperactivate mTOR, fuelling glycolytic reprogramming and aggressive invasion; conversely, in colorectal cancer, VSIG2 loss is linked to reduced M1 macrophage and B-cell infiltration, dampened antigen presentation, and shortened survival [ 8, 9]. Single-cell interrogations further revealed that VSIG2 expression inversely correlates with cytotoxic CD8⁺ T-cell programs and synergizes with tumour-derived lactate to enforce an immunosuppressive niche [10]. However, whether VSIG2 plays a critical role in GC progression and is a tractable target for next-generation precision immunotherapy remain to be probed.

K63-linked ubiquitination has emerged as a non-proteolytic “signal code” that dictates the timing and strength of innate immune and DNA damage responses [11]. Unlike canonical K48 chains, K63 polymers assemble as flexible scaffolds that recruit adaptors—such as TAB2/3, NEMO or OPTN—into dynamic signaling hubs rather than delivering substrates to the proteasome [ 12– 14]. Recent structural snapshots revealed that the E2-E3 pair Ubc13/Uev1A-TRAF6 forms a catalytic filament on the membrane-proximal surface of TLR4 endosomes, thereby facilitating rapid K63 chain growth in response to minute amounts of LPS [15]. Intriguingly, K63 chains can coexist with K48 or M1 linkages in heterotypic or branched architectures, enabling cells to fine-tune NF-κB activation, phagosome maturation or cGAS-STING signaling without triggering premature cell death [16]. Understanding how these “mixed” topologies are written, read and erased is now a central challenge in immunometabolism and cancer biology.

In this study, we aimed to determine the role of VSIG2 in GC progression. Our results reveal that low VSIG2 levels in GC drive the proliferation and migration of cancer cells. Mechanistically, VSIG2 inhibits the activation of the NF-κB pathway by suppressing FBXW10-mediated K63 ubiquitination of ANXA2. This study elucidates a new mechanism by which VSIG2 inhibits GC progression, providing a new perspective for the diagnosis and treatment of GC.

## Materials and Methods

### Clinical samples and cell lines

Gastric cancer (GC) specimens were procured from patients who underwent radical gastrectomy at the Department of Gastrointestinal Surgery, the Affiliated Hospital of Nantong University (Nantong, China). Participants did not receive adjuvant chemotherapy prior to their surgery. The collection of GC tissue samples spanned from November 2016 to October 2017. All participants provided informed consent, and the study was conducted in compliance with the Declaration of Helsinki principles and was granted approval by the Ethics Committee of the Affiliated Hospital of Nantong University under the reference number (2014-L103). Five GC cell lines (AGS, HGC27, MKN-45, MKN-28, and NCL-N87), and a normal human gastric epithelial cell line (GES-1) were obtained from the Shanghai Institutes for Biological Sciences (Shanghai, China). The HGC-27, MKN-45, MKN-28, and NCL-N87 cell lines were grown in Roswell Park Memorial Institute 1640 (RPMI-1640; Gibco, Carlsbad, USA) medium, while the AGS cells were maintained in Kaighn’s Modified Ham’s F-12K (F12K; Gibco) medium. Each type of medium was supplemented with 10% fetal bovine serum (FBS; Wisent, Saint-Jean-Baptiste, Canada) and 1% penicillin/streptomycin (Gibco). Mycoplasma testing was performed using a Venor® Gem qEP kit (Minerva Biolabs, Berlin, Germany) at 3-month intervals to maintain cell line integrity and purity.

### Quantitative real-time polymerase chain reaction (qRT-PCR)

Total RNA was recovered with a silica-membrane spin column (EZ-10 Mini-Prep; Sangon Biotech, Shanghai, China) pre-treated with DNase I to eliminate genomic traces. An RNA integrity number (RIN) ≥ 8.3 was confirmed on a Fragment Analyzer before mass was equalized across samples to 500 ng. Reverse transcription was carried out in 10 μL reactions containing anchored oligo-dT and gene-specific primers ( Supplementary Table S1) using LunaScript RT Mix (NEB, Beverly, USA) at 55°C for 10 min; the cDNA was diluted 1:5 and stored at −80°C. The target transcripts were quantified by qPCR and normalized to the geometric mean of *GAPDH* expression.

### Lentivirus and transfection

To establish stable cell lines, cells were subjected to transfection via lentiviral vectors carrying either a negative control or short hairpin RNA constructs (GenePharm, Shanghai, China) designed to target VSIG2. The transfection process included the use of polybrene (at a concentration of 5 mg/mL; Sigma-Aldrich, St Louis, USA) to enhance efficiency, at a multiplicity of infection of 10. At 72-h post-transfection period, the selection of stable cell lines was initiated through treatment with puromycin (at a concentration of 10 μg/mL) for three consecutive days. Flag-tagged, His-tagged and HA-tagged expression vectors and mutants were sourced from GenePharm (Shanghai, China). Small interfering RNAs and plasmids were transfected into cells using Lipofectamine3000 (Invitrogen) in strictly accordance with the manufacturer’s protocol.

### Western blot analysis

Tissues or cells were homogenized in 300 μL of a non-denaturing lysis cocktail (50 mM Tris-HCl pH 7.4, 150 mM KCl, 1 mM EDTA, and 1% n-dodecyl-β-D-maltoside) supplemented with cOmplete™ EDTA-free protease inhibitor tablets (Roche, Basel, Switzerland). The lysates were clarified via two-step centrifugation (500 *g*, 5 min, 4°C followed by 16,000 *g*, 15 min, 4°C) to eliminate nuclei and debris. The protein concentration was determined using a micro-BCA assay kit (Pierce, Rockford, USA) in a 2-μL sample volume. Equalized lysates (25 μg) were fractionated on 4%–20% precast gradient mini-gels (Bio-Rad TGX Stain-Free; Bio-Rad Laboratories, Hercules, USA) and transferred onto low-fluorescence 0.2 μm PVDF membranes activated in 100% methanol for 20 s. Membranes were blocked for 30 min in 5% fish gelatin dissolved in TBST plus 0.05% Tween-20 and then probed overnight at 4°C with the following primary antibodies: anti-VSIG2 (PA5-62144; Thermo Fisher Scientific, Waltham, USA), anti-ANXA2 (60051-1-Ig; Proteintech, Rosemont, USA), anti-K63 Ub (D7A11, Cell Signaling Technology, Inc., Danvers, USA), anti-Ub (A19686, Abcolonal, Wuhan, China), anti-FBXW10 (PA5-43814;, Thermo Fisher Scientific) and GAPDH (10494-1-AP; Proteintech). Bound antibodies were detected with species-specific HRP-conjugated secondary antibodies (MultiSciences, Hangzhou, China) and enhanced chemiluminescence reagent (SuperSignal™ Pico Plus; Thermo Fisher Scientific).

### Immunohistochemistry (IHC)

IHC was performed on 4-μm paraformaldehyde-fixed, paraffin-embedded sections mounted on positively charged, low-autofluorescence glass slides (SuperFrost Plus-white; Thermo Fisher Scientific). Deparaffinization was performed using a three-solvent gradient (xylene-free citrus substitute, 3 min; 100% ethanol, 2 min; 70% ethanol, 1 min) followed by proprietary deparaffinization of oil (Clearify™; Sakura, Tokyo, Japan) to eliminate residual lipids. Antigen retrieval was performed in a pressure-controlled microwave (10 psi, 110°C, 5 min) using pH 9.0 borate-TEA buffer supplemented with 0.05% Tween-20 and 1 mM EDTA to increase epitope exposure while preventing calcium-mediated artefactual precipitation. Endogenous peroxidase was quenched with 3% H _2_O _2_ in methanol for 8 min at 4°C; endogenous biotin was then saturated by sequential incubation with streptavidin (15 min) and biotin (15 min) (Vector SP-2001). The sections were blocked for 30 min at 20°C in 10% donkey serum, 2% BSA, 0.3% fish gelatin and 0.1% saponin to minimize the hydrophobic and ionic background. The primary rabbit monoclonal antibody (clone EP282Y, 1:400; Abcam, Cambridge, UK) was diluted in the same blocking buffer and incubated overnight at 4°C under humidified parafilm coverslips to prevent evaporation. An HRP-conjugated, cross-species-adsorbed secondary antibody (1:500; donkey anti-rabbit IgG-HRP, Jackson 711-035-152; West Grove, USA) was used for 45 min at 20°C, followed by incubation with 3,3′-diaminobenzidine (DAB)-H _2_O _2_ substrate (Vector SK-4105) developed for exactly 90 s under a calibrated Lux meter (150 lx) to ensure run-to-run consistency. The slides were counterstained with 0.02% toluidine blue for 45 s to enhance nuclear morphology, dehydrated through a graded alcohol series, cleared with limonene-based mountant (Cytoseal 60) and coverslipped. The staining intensity (0–3+) and percentage positive area were quantified via QuPath v0.5 with a custom random-forest classifier trained on > 3000 manually annotated pixels per slide.

### Immunofluorescence staining

Immunofluorescence labelling was performed on cells grown on 12 mm, #1.5H borosilicate coverslips pre-coated with 0.5 μg cm ^-^² laminin-521 in carbonate buffer (pH 9.4, 4°C, 16 h) to minimize focal-adhesion artefacts. After fixation with 2% glyoxal in pHEM buffer (pH 6.9) for 7 min at 37°C, the cells were permeabilized for 90 s with 0.1% saponin + 0.02% digitonin in ice-cold PBS to create discrete plasma-membrane pores without nuclear leakage. Blocking was carried out for 30 min at 20°C in “BlockAid” solution (5% normal donkey serum, 1% cold-water fish gelatin, 0.05% casein, and 0.01% Tween-20) to suppress both hydrophobic and charge-based non-specific binding. Primary antibodies were diluted in blocking buffer containing 2 mM sodium azide to prevent microbial growth, and coverslips were inverted onto 20 μL antibody droplets sealed under mineral oil to eliminate evaporation artefacts. Following overnight incubation at 4°C, the samples were washed three times (2 min each) in custom high-salt PBS (500 mM NaCl + 0.1% Tween-20) to strip loosely bound IgG. The secondary antibodies (cross-adsorbed, 1:750) were conjugated to either the CF680R or STAR RED dyes and incubated for 45 min at 20 °C in the dark. After the final washes, the DNA was counterstained with 0.5 μg mL ^-^¹ SiR-Hoechst for 10 min, and the coverslips were mounted in 90% glycerol + 0.5% n-propyl gallate + 0.01% PVA to reduce refractive index mismatch. Imaging was performed on a spinning-disk confocal system (60×, 1.27 NA water objective) with adaptive optics; Z-stacks (0.12 μm step) were deconvolved using Huygens PSF-distilled kernels and analyzed in Imaris 10.1 with automated colocalisation thresholding.

### Co-immunoprecipitation (co-IP) assay

Co-IP was carried out via a membrane-restricted, detergent-swap protocol to preserve weak and transient ANXA2-VSIG2 complexes. The cells (2 × 10 ^7^) were rinsed twice in ice-cold PBS-Ca ^2+^/Mg ^2+^ and cross-linked with 2 mM dithiobis (succinimidyl propionate; DSP) in 20 mM HEPES, pH 8.0, for 30 min at 4°C. Cross-linking was quenched with 50 mM Tris-HCl, pH 7.4, for 15 min. Cells were then lysed in a two-step buffer series: first, 0.5% digitonin in 150 mM NaCl, 50 mM Tris-HCl, pH 7.4, and 1 mM CaCl _2_ (10 min, 4°C) to solubilize cholesterol-rich microdomains; second, CHAPS (final 0.3%) was added for an additional 5 min to release nonlipid raft proteins. The lysates were cleared at 20,000 *g* for 5 min at 4°C and pre-cleared with 20 μL of Protein A/G PLUS-Agarose (Santa Cruz Biotechnology, Santa Cruz, USA) for 30 min. The precleared lysates were incubated overnight at 4°C with 2 μg of rabbit anti-ANXA2 conjugated to NHS-activated magnetic beads (Dynabeads™ M-270 Epoxy; Life Technologies, Carlsbad, USA) that had been pre-blocked with 0.5% fish gelatin. The beads were washed three times in high-stringency buffer (500 mM NaCl, 0.1% CHAPS, and 10 mM Tris-HCl, pH 7.4) and once in 50 mM ammonium bicarbonate to remove residual detergent. The captured proteins were eluted by heating at 95°C for 5 min in 1× lithium dodecyl sulfate sample buffer containing 50 mM DTT. Eluates were analyzed by western blot analysis or subjected to LC-MS/MS for interactome mapping. We entrusted BGI (Beijing Genomics Institute, Beijing, China) with the responsibility of conducting mass spectrometry analysis on the test samples.

### Cell counting kit-8 (CCK-8) assay

A total of 1 × 10³ cells per well were seeded in 96-well, ultraclear-bottom plates (#3904; Corning, New York, USA) pre-coated with 0.15 mg/mL synthetic basement-membrane hydrogel to mimic tumor stiffness. After 24 h of incubation at 37°C, 5% CO _2_, and 95% humidity, the medium was exchanged for 90 μL of antibiotic-free, phenol red-free RPMI-1640 plus 10 mM HEPES to buffer pH drift. Ten microlitres of WST-8 reagent (CK04-20; Dojindo, Tokyo, Japan) were gently layered on top using a reverse-pipetting technique to avoid shear stress. The plate was transferred to a custom, sealed micro-incubator cassette (IncuCyte S3; Sartorius, Shanghai, China) and incubated for exactly 2 h while maintaining at 37.0 ± 0.1°C and 5.0% ± 0.1% CO _2_. The absorbance was recorded at 450 nm with a 620 nm reference filter.

### 5-Ethynyl-2′-deoxyuridine (EdU) assay

For EdU pulse-labelling, cells (8 × 10³ per well) were seeded in 96-well plates pre-coated with 0.1% poly-L-lysine. After 24 h, 10 μM 5-ethynyl-2′-deoxyuridine (EdU, Click-iT™ EdU; Thermo Fisher Scientific) was added, and the mixture was incubated for exactly 45 min at 37°C under 5% CO _2_. Fixation was performed with 3% glyoxal in PBS (pH 7.2) for 8 min at 20°C, followed by 0.1% Triton X-100 permeabilisation for 90 s. The click reaction was run for 15 min in the dark with 0.2 mM CuSO _4_ and 2 mM ascorbic acid. Nuclei were counterstained with 1 μg/mL Hoechst 33342 for 5 min. Images were acquired on an ImageXpress Micro Confocal system (20×, 0.75 NA) and analyzed via MetaXpress integrated intensity segmentation. EdU-positive nuclei are expressed as a percentage of total nuclei per field.

### Transwell migration assay

Cell migration was quantified in 24-well FluoroBlok inserts (8 μm pores; Corning). A total of 2 × 10 ^4^ serum-starved cells in 200 μL of 1640 with 0.1% FBS were seeded into the inserts; 750 μL of complete medium served as a chemoattractant. After 36 h at 37°C and 5% CO _2_, the inserts were fixed in 3% glyoxal (5 min) and stained with 2 μM calcein-AM for 30 min. Migratory cells on the underside were counted in three random fields.

### Wound healing assay

A wound healing assay was performed in 24-well plates. Upon reaching 95% confluence, the cells were serum-starved for 6 h, and a 600-μm wide, debris-free gap was created via a sterile 200 μL pipette tip. The plates were rinsed twice with PBS + 2 mM Ca²⁺ to remove cellular debris and then incubated in 1% FBS medium to suppress proliferation. Phase-contrast images were captured at 24 h with a Nikon microscope (Tokyo, Japan), and the wound area was quantified using ImageJ software.

### Mouse models

Four-week-old male BALB/c nude mice ( *n* = 5) were acclimatized for 7 days in individually ventilated cages (22°C, 50% humidity, 12/12 h light/dark cycle). For the xenograft tumor model, a 100-μL suspension containing 2 × 10 ^6^ tumor cells was injected subcutaneously into the dorsal flank. Tumor volume was calculted as follows: tumor volume = (length × width²)/2. The mice were euthanized via CO _2_ asphyxiation followed by cervical dislocation at the endpoint (day 28). Tumors were excised and snap-frozen in liquid N _2_. For the liver metastasis model, a 0.8 cm skin incision was made at the left flank, followed by blunt dissection to exteriorize the spleen. A total of 5 × 10 ^4^ tumor cells in 30 μL of PBS were slowly injected into the splenic pulp via a 33 G Hamilton syringe. After 3 min, the spleen was gently returned, the peritoneum was closed with 6-0 absorbable sutures, and the skin was sealed with tissue adhesive. On day 28, under terminal anaesthesia, the livers were harvested for *ex vivo* imaging and histology. All protocols were approved under IACUC #2025-07-05.

### Statistical analysis

Every dataset was generated from a minimum of three biologically independent runs, each performed in triplicate. Central tendency and dispersion are reported as the mean ± SD after outlier exclusion via the robust ROUT method. Normality was first interrogated with the Shapiro-Wilk omnibus test; variables deviating from Gaussian distributions were analyzed with the two-tailed Mann-Whitney U or Kruskal-Wallis test with Dunn’s correction, whereas parametric equivalents employed Welch-corrected unpaired *t* tests or Brown-Forsythe ANOVA. The contingency between VSIG2 status, ANXA2 status and clinicopathologic features was assessed with Fisher’s exact test when the expected count fell below five; otherwise, the χ² test was used. Survival curves were constructed via the Kaplan–Meier estimator, with hazard differences evaluated by the Mantel-Cox log-rank test and Harrell’s C-index for model discrimination. A *P* value less than 0.05 was considered as the threshold for statistical significance. All computations were executed in GraphPad Prism 10 (San Diego, USA).

## Results

### Epithelial cell-derived VSIG2 is downregulated in GC and is negatively associated with prognosis.

**Figure 1:**
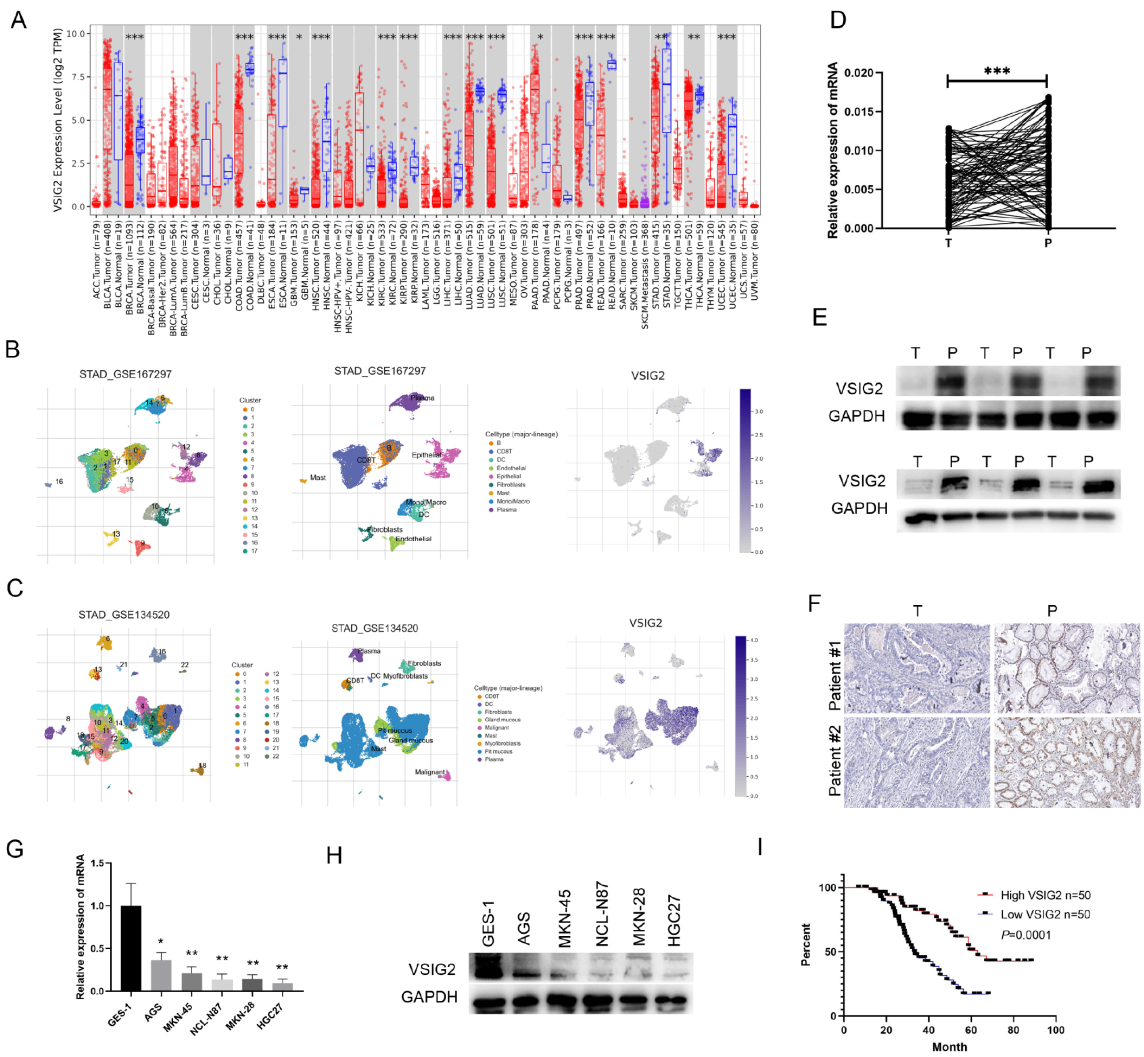
Figure 1 Epithelial cell-derived VSIG2 is downregulated in GC and is negatively associated with prognosis (A) VSIG2 expression in multiple cancers was analysed via the TCGA database. (B,C) VSIG2 expression in different cell subsets was analyzed based on GEO database. (D) The mRNA expressions of VSIG2 in GC and paired adjacent tissues were detected by RT-qPCR. (E,F) The protein levels of VSIG2 in GC and paired adjacent tissues were detected by (E) western blot analysis and (F) immunohistochemistry. (G) VSIG2 mRNA s in GC cell lines and normal endothelial cells were detected by RT-qPCR. (H) The protein levels of VSIG2 in GC cell lines and normal endothelial cells were detected by western blot analysis. (I) Kaplan-Meier curves for the overall survival (OS) of GC patients with high VSIG2/low VSIG2 expression levels were generated via RT-qPCR. *P < 0.05, **P < 0.01, ***P < 0.001.

To explore the key molecules that regulate the progression of GC, we integrated and analyzed data from the TCGA and GEO databases (GSE26942, GSE27342 and GSE163558) and found that VSIG2 was significantly differentially expressed (|log2(fold change)| ≥ 1 and *P*  < 0.05) in GC tissues ( Figure 1A). Single-cell data mining revealed that VSIG2 was expressed mainly in epithelial cells ( Figure 1B), and further exploration revealed that VSIG2 was localized mainly in the mucosal epithelium rather than in malignant epithelial cells ( Figure 1C). Given that, we then performed PCR detection on GC samples from our hospital. The results revealed that the expression of VSIG2 in cancer tissues was significantly lower than that in adjacent tissues ( Figure 1D). Subsequently, we randomly selected cancer tissues and adjacent tissues from 6 patients and performed protein-level detection. Consistent with previous data, the expression level of VSIG2 in cancer tissues was significantly lower than that in adjacent tissues ( Figure 1E). The results of the immunohistochemical staining also confirmed these findings ( Figure 1F). We subsequently detected the expression level of VSIG2 in gastric epithelial cells and GC cells. The results revealed that the level of VSIG2 in gastric cancer cell lines was significantly lower than that in normal gastric epithelial cells ( Figure 1G,H).

To explore the relationship between VSIG2 expression and clinical prognosis, we performed correlation analysis on the basis of the PCR results. We found that patients with low VSIG2 levels were more likely to have lymph node metastasis, vascular invasion, high TNM stage and larger tumors ( Table 1). Further analysis revealed that patients with low VSIG2 expression had a worse prognosis ( Figure 1I). Together, the results demonstrated that VSIG2 might be a competent indicator of GC progression and patient survival.

**Table:** **Table 1** Relationship between VSIG2 expression and clinico-pathological features in GC patients ( *n* = 100)

Characteristics	VSIG2				*P*
	Low expression ( *n* = 50)	High expression ( *n* = 50)	Total		
Age					
< 60	24	23	47		0.841
≥ 60	26	27	53		
Gender					
Male	24	28	52		0.423
Female	26	22	48		
Tumor size					
< 3cm	21	33	54		0.016*
≥ 3cm	29	17	46		
Tumor site					
Proximal	32	25	57		0.157
Non-proximal	18	25	43		
Lymph node metastasis					
N0	16	29	45		0.009*
N1–N3	34	21	55		
TNM stage					
I–II	15	27	42		0.015*
III	35	23	58		
Vascular invasion					
Negative	10	22	32		0.010*
Positive	40	28	68		

### VSIG2 inhibits GC cell progression *in vitro*

**Figure 2:**
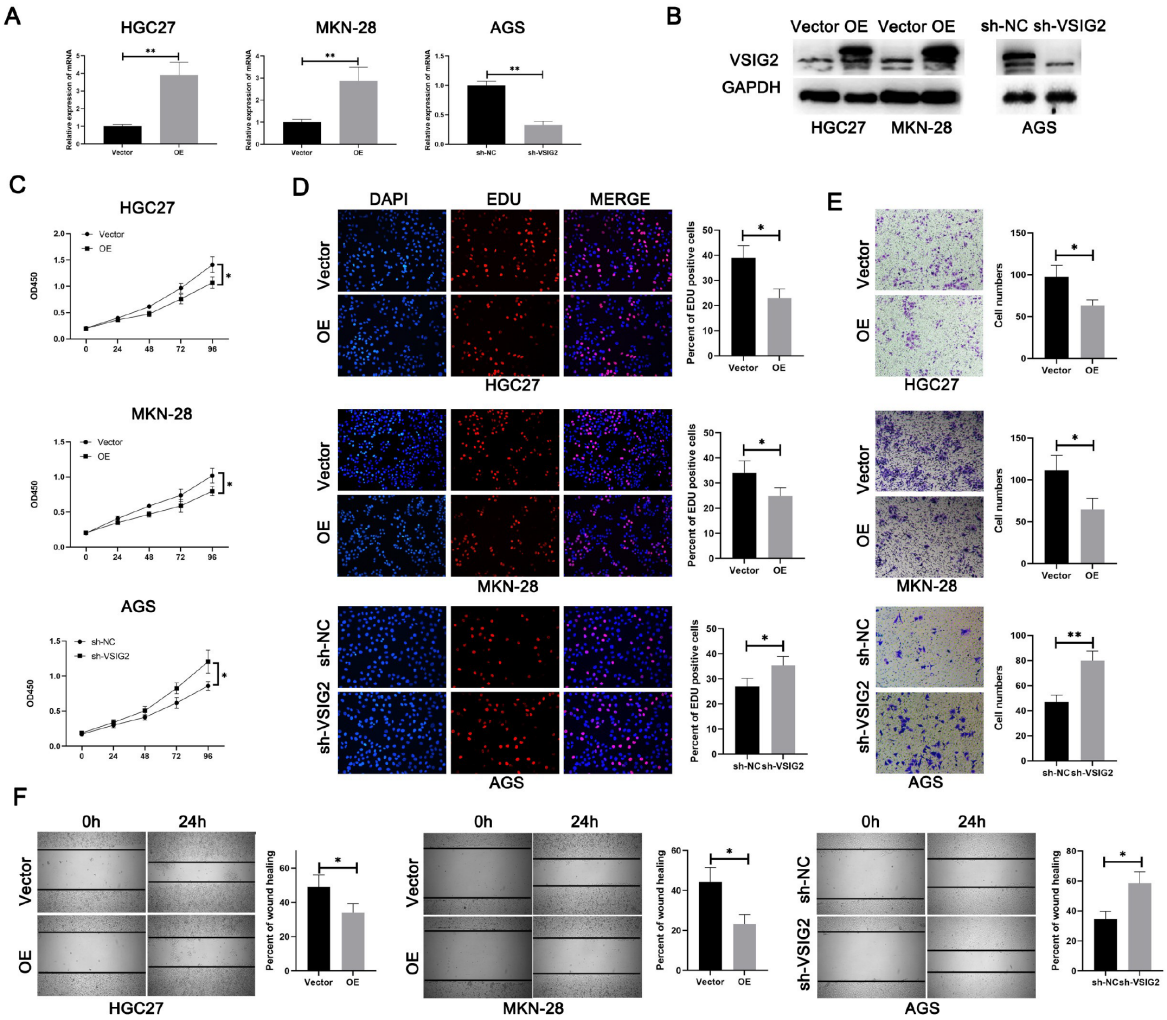
Figure 2 VSIG2 inhibits GC cell progression *in vitro* (A,B) The transfection efficiency of VSIG2 was verified by (A) qRT-PCR and (B) western blot analysis. (C–F) The effects of VSIG2 on GC cells were evaluated based on (C) cell growth assay, (D) EdU incorporation assay, (E) Transwell assay and (F) wound healing assay. *P < 0.05, **P < 0.01.

To further explore the role of VSIG2, we selected HGC27 and MKN-28 cells with relatively low expression of VSIG2 for overexpression and AGS cells with relatively high expression of VSIG2 for knockdown. The transfection efficiency was then verified ( Figure 2A,B). We used the constructed cell line for subsequent cell function and mechanism exploration experiments.

First, we examined the effect of VSIG2 on the proliferation of GC cells via a CCK8 assay. The results of growth curve analysis revealed that the growth rate of HGC27 and MKN-28 cells after VSIG2 overexpression was significantly lower than that of the control cells ( Figure 2C), whereas VSIG2 silencing significantly increased the growth rate of AGS cells. Next, we performed an EDU experiment. The results revealed that the DNA synthesis ability of HGC27 and MKN-28 cells in the overexpression group was significantly lower than that in the control group. However, after *VSIG2* was knocked down in AGS cells, the DNA synthesis ability of GC cells was significantly enhanced ( Figure 2D). Taken together, these experimental results indicate that VSIG2 can weaken the proliferative ability of tumor cells in GC.

GC is a highly invasive tumor, making it easier to metastasize through blood vessels and the lymphatic system into other organs. Therefore, we explored the effect of VSIG2 on GC migration and metastasis. First, the effect of VSIG2 on the migration ability of GC cells was detected via a Transwell assay. The results revealed that the number of cells that passed through the chamber in the VSIG2-overexpressing group was significantly lower than that in the control group. The number of AGS cells with *VSIG2* knockdown that passed through the chamber was significantly higher than that in the control group ( Figure 2E). The scratch test results revealed that, compared with that of the control group, the migration distance of HGC27 and MKN-28 cells overexpressing VSIG2 was significantly shorter within 24 hours. In contrast, after *VSIG2* was silenced, the migration distance of AGS cells within 24 h was significantly greater than that of the control group ( Figure 2F). Taken together, the above results indicate that VSIG2 can inhibit the proliferation and migration of GC cells *in vitro*.

### VSIG2 hinders GC progression *in vivo*

**Figure 3:**
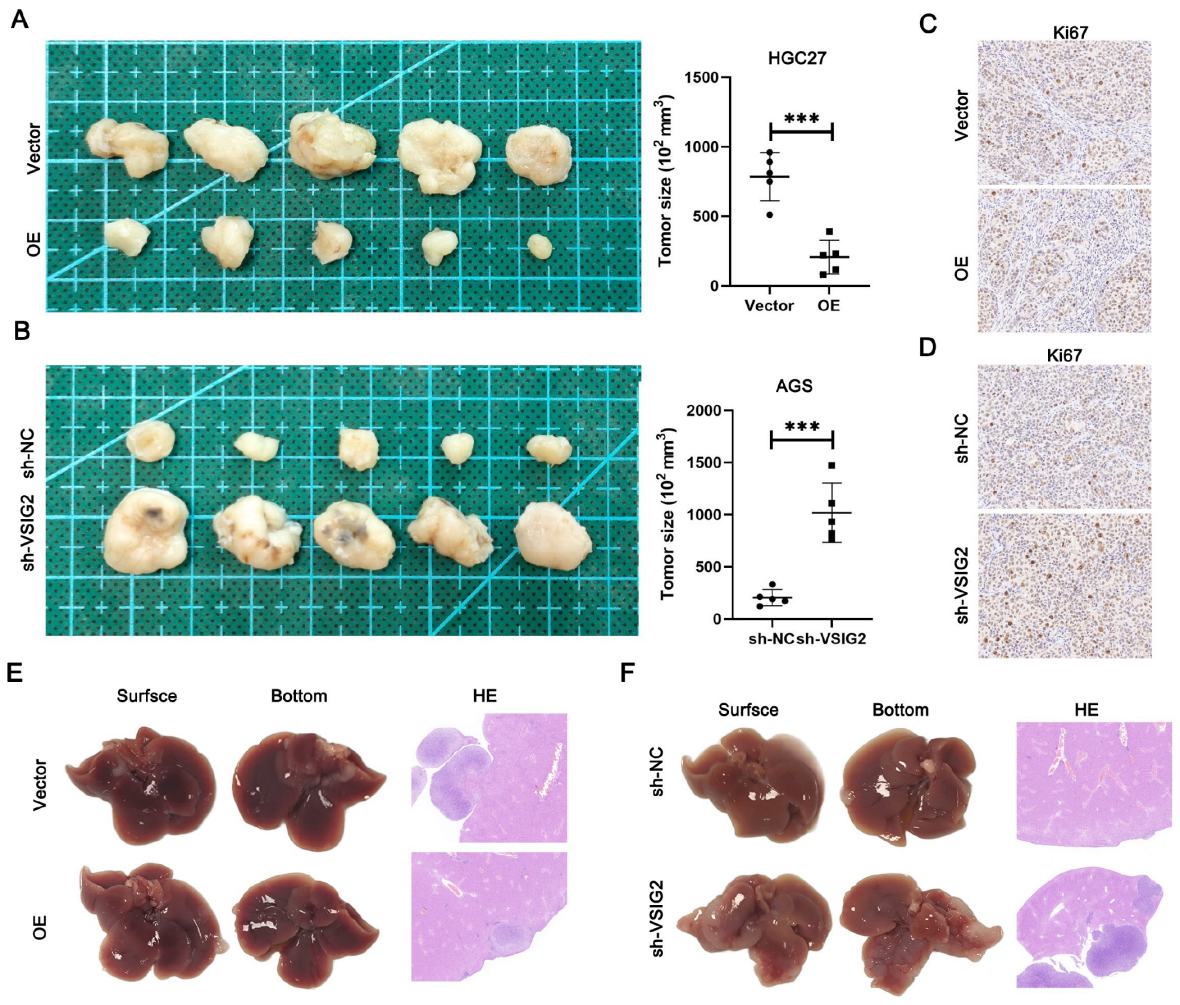
Figure 3 VSIG2 hinders GC progression *in vivo* (A,B) Representative images showing tumors removed from nude mice subcutaneously injected with VSIG2-overexpressing HGC27 cells or VSIG2-silenced AGS cells and quantification of tumor size (n = 5). (C,D) Representative images of IHC staining for ki67 in nude mouse-derived tumors in which VSIG2 was knocked down or overexpressed. (E,F) Representative images showing livers removed from mice intrasplenicly injected with VSIG2-overexpressing HGC27 cells or VSIG2-silenced AGS cells and the corresponding HE-stained samples. ***P < 0.001.

To further study the effect of VSIG2 on the progression of GC, we designed a tumor formation experiment in nude mice. HGC27 cells with VSIG2 overexpression or not and AGS cells with *VSIG2* knockdown or not were injected subcutaneously into nude mice to construct a subcutaneous tumor formation experiment in nude mice. Compared with that of the control group, the tumor volume of HGC27 cells overexpressing VSIG2 in nude mice was significantly reduced ( Figure 3A). In contrast, compared with that of AGS cells transfected with the empty vector, the subcutaneous tumor volume of AGS cells with *VSIG2* knockdown was significantly greater ( Figure 3B). We subsequently collected subcutaneous tumors from the nude mice in each group for Ki67 immunohistochemical staining. The results revealed that the expression of Ki-67 in the subcutaneous tumors of the nude mice in the VSIG2-overexpressing group was lower than that in the control group. However, Ki-67 protein expression in the subcutaneous tumors of the nude mice in the *VSIG2*-knockdown group was significantly greater than that in the corresponding control group ( Figure 3C,D). These results suggest that VSIG2 inhibits the proliferation of GC *in vivo*.

To further explore the ability of VSIG2 to affect the invasion and metastasis of GC cells *in vivo*, we further injected HGC27 cells and AGS cells into the spleens of each group of nude mice to construct an intrahepatic metastasis model. Compared with those in the HGC27-Vector group, the symptoms of intrahepatic metastases formed in the HGC27-OE group were significantly lower, and the degree of cancer cell infiltration into intrahepatic metastases shown by HE staining was also significantly lower ( Figure 3E). In contrast, the symptoms of intrahepatic metastases formed in the AGS-sh-VSIG2 group were significantly greater than those in the AGS-sh-NC group, and the degree of cancer cell infiltration into intrahepatic metastases shown by HE staining was significantly greater ( Figure 3F). These results suggest that VSIG2 inhibits GC proliferation and metastasis *in vivo*.

### VSIG2 interacts with ANXA2 and induces its membrane location.

**Figure 4:**
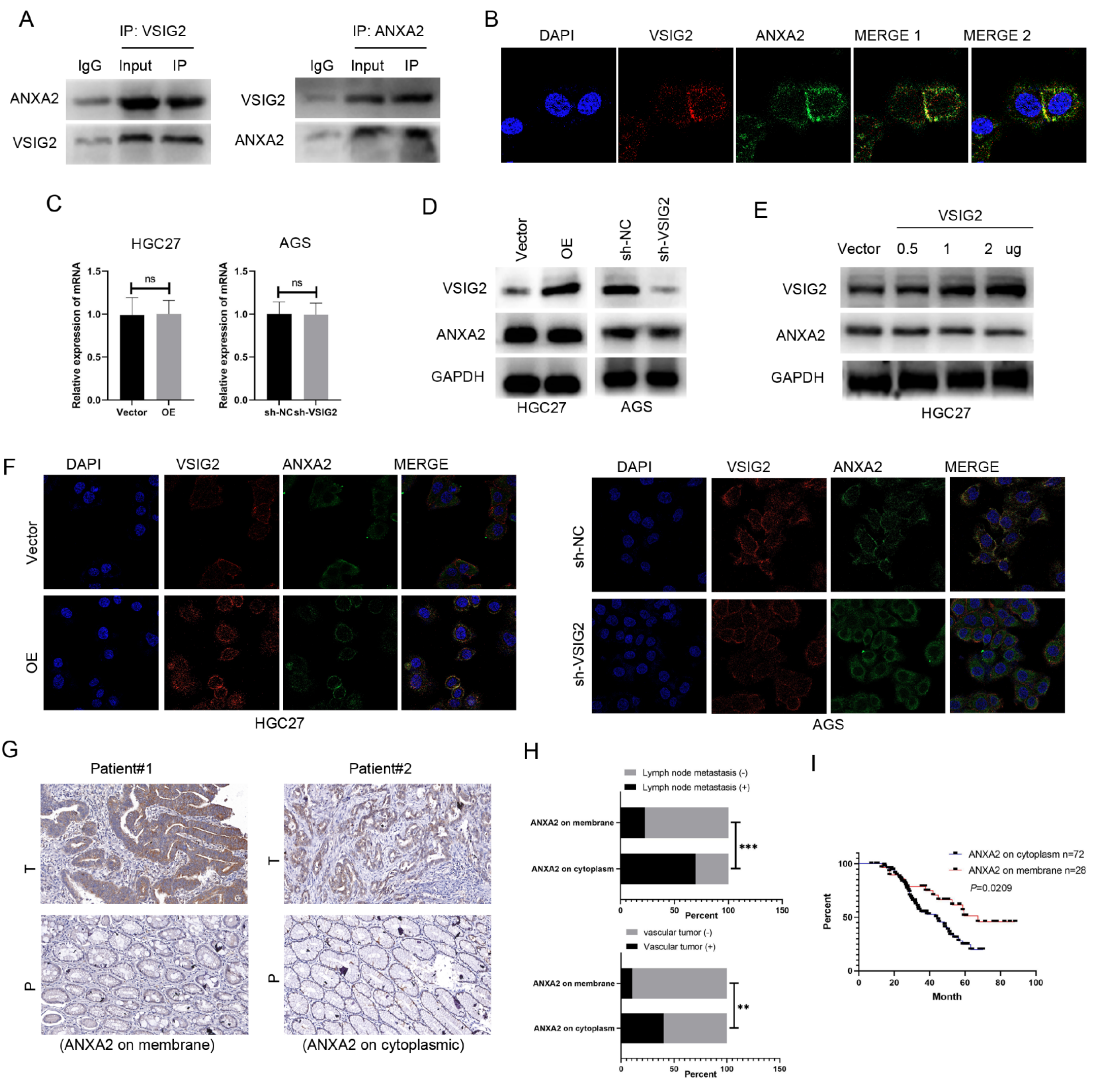
Figure 4 VSIG2 interacts with ANXA2 and induces its membrane location (A,B) Co-IP and immunofluorescence were performed to determine the interaction between VSIG2 and ANXA2. (C,D) The expression of ANXA2 in GC cells with VSIG2 knockdown or overexpression was detected via qRT-PCR and western blot analysis. (E) ANXA2 expression in GC cells treated with increasing doses of VSIG2 was detected via western blot analysis. (F) The subcellular localization of ANXA2 with VSIG2 knockdown or overexpression was detected. (G) Representative images of IHC staining for ANXA2 on the membrane or in the cytoplasm. (H,I) Relationship between ANXA2 on the cytoplasm/ANXA2 on the cell membrane and clinical correlation and prognosis. **P < 0.01, ***P < 0.001.

To further explore the potential mechanism of VSIG2 in GC development, we isolated proteins that interact with VSIG2 in cells. By selecting the AGS cell line and extracting its protein via the VSIG2 antibody, we used the Co-IP method to pull down the proteins that may bind to VSIG2 and then performed mass spectrometry (LC-MS/MS) analysis. Among the top 10 binding proteins of coverage, we identified the peptide segment of ANXA2. ANXA2 is a calcium-mediated phospholipid-binding protein that is widely expressed in various tissues and organs of mammals and can stimulate the metastasis, invasion and proliferation of various types of tumors, including GC [ 17– 20]. Recently, ANXA2 was shown to play an important role in regulating the expression of tumor-associated fibroblasts (CAFs) and infiltrating immune-related cells, as well as in the synergy between endothelial cells and the tumor microenvironment (TME) [21]. To verify the mass spectrometry data, we performed Co-IP experiments with VSIG2 and ANXA2 antibodies in AGS cell lines. The results revealed that VSIG2 interacted with ANXA2 ( Figure 4A). Next, we performed immunofluorescence experiments in the AGS cell line to further explore the spatial co-localization of the two proteins. The results revealed that VSIG2 and ANXA2 were co-localized in the cell membrane ( Figure 4B). We subsequently aimed to verify the significance of the interaction between VSIG2 and ANXA2. PCR and western blot analysis results revealed that the expression level of ANXA2 did not change significantly regardless of high VSIG2 expression or low VSIG2 expression ( Figure 4C,D). Further detection revealed that the expression level of ANXA2 did not change with increasing VSIG2 expression ( Figure 4E), suggesting that VSIG2 affects the spatial localization of ANXA2. Immunofluorescence revealed that after VSIG2 overexpression, ANXA2 was significantly located in the cell membrane. In contrast, when *VSIG2* was knocked down, the cytoplasmic localization of ANXA2 was significantly increased ( Figure 4F), which was consistent with our hypothesis. To further study the correlation between the spatial localization of ANXA2 and clinical patients, we performed IHC staining of a tissue microarray (TMA). We selected samples with typical cytoplasmic expression of ANXA2 and cell membrane expression of ANXA2 for immunohistochemistry ( Figure 4G). Analysis of the TMA revealed that patients with cytoplasmic ANXA2 expression were more likely to have lymph node metastasis and vascular invasion ( Figure 4H). Prognostic analysis revealed that the prognosis of patients with cytoplasmic ANXA2 expression was significantly worse than that of patients with normal ANXA2 expression ( Figure 4I). On the basis of the above data, the VSIG2/ANXA2 complex may play an important role in GC.

### VSIG2 competes with FBXW10 for binding to ANXA2 and relies on FBXW10-mediated K63 polyubiquitination of ANXA2

**Figure 5:**
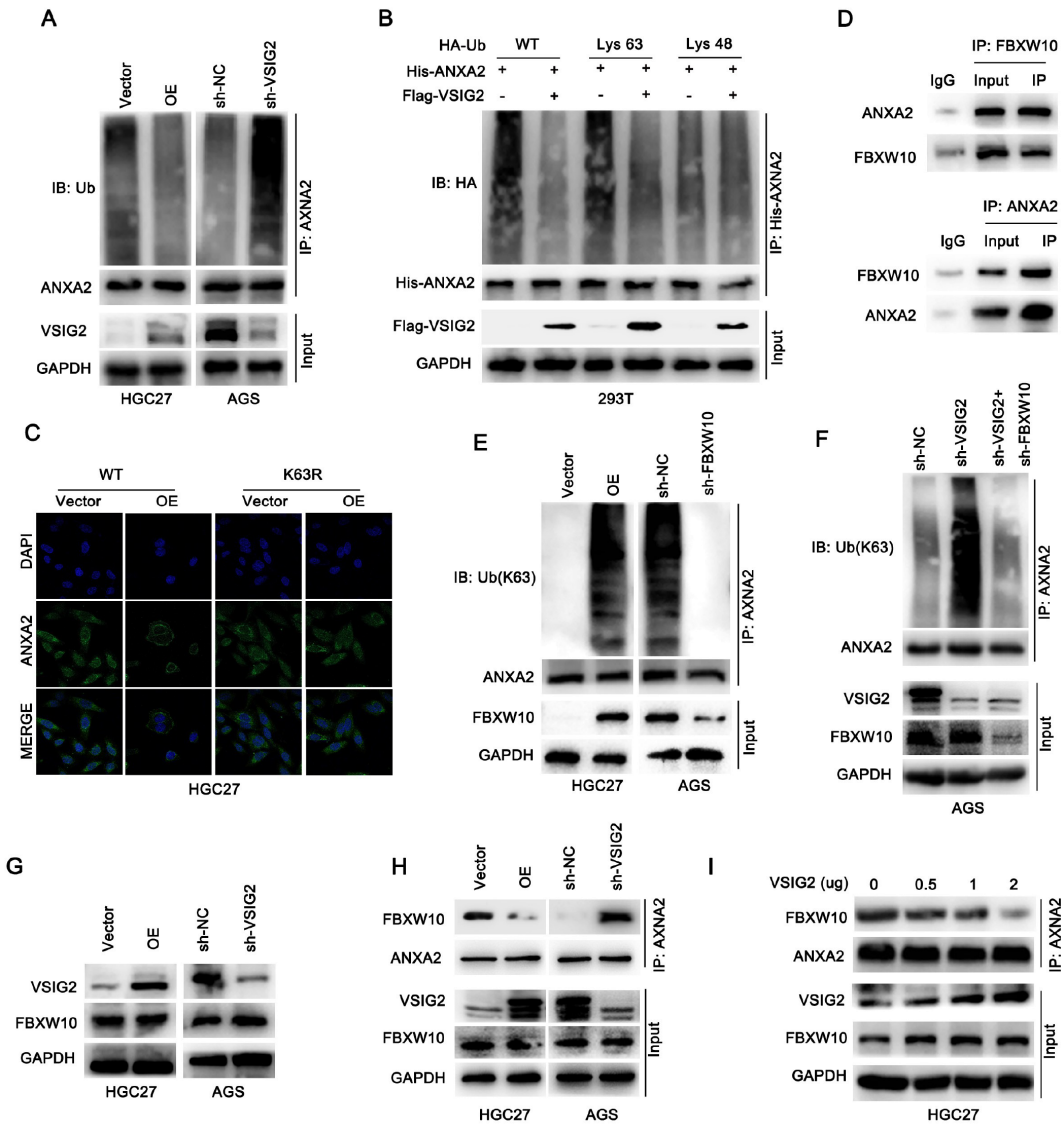
Figure 5 VSIG2 competes with FBXW10 for binding to ANXA2 and relies on FBXW10-mediated K63 polyubiquitination of ANXA2 (A) ANXA2 ubiquitination was analyzed in GC cells with VSIG2 knockdown or overexpression. (B) HA-tagged Ub-WT, Ub-K48, and Ub-K63 mutant plasmids, His-tagged ANXA2 plasmid and Flag-tagged VSIG2 plasmid were transfected into 293T cells. After 48 h of culture, protein was extracted, immunoprecipitation was performed with His-ANXA2 antibody, and HA was detected by immunoblotting. (C) The subcellular localization of ANXA2 at K63R was detected. (D) Co-IP was performed to analyze the interaction between FBXW10 and ANXA2. (E) ANXA2 K63 ubiquitination was analyzed in GC cells with FBXW10 knockdown or overexpression. (F) ANXA2 K63 ubiquitination levels were analyzed in GC cells with VSIG2 knockdown alone or with VSIG2 knockdown combined with FBXW10 knockdown. (G) The expression level of FBXW10 with VSIG-knockdown or overexpressing cells was detected. (H) The binding affinity of the FBXW10/ANXA2 complex was detected in GC cells with VSIG2 knockdown or overexpression. (I) The binding affinity of the FBXW10/ANXA2 complex in GC cells with increasing doses of VSIG2 was detected.

Considering that a change in the VSIG2 level does not affect the expression level of ANXA2, we speculate that the change in ANXA2 localization may be related to VSIG2-mediated post-translational modification. Previous studies have shown that ubiquitination not only changes protein expression levels but also changes protein localization and affects signal transduction. Therefore, we first examined the effects of different VSIG2 expression levels on ANXA2 ubiquitination. Consistent with our hypothesis, VSIG2 overexpression significantly decreased the level of ANXA2 ubiquitination, whereas *VSIG2* knockdown significantly increased the level of ANXA2 ubiquitination ( Figure 5A). K48- and K63-dependent ubiquitination is the most common form of modification. We found that VSIG2 changed mainly the K63 ubiquitination level of ANXA2 ( Figure 5B). Further analysis revealed that after the K63 mutation, the membrane localization of ANXA2 mediated by VSIG2 became cytoplasmic. The above results revealed that the change in the spatial localization of ANXA2 mediated by VSIG2 depended on its K63-mediated ubiquitination ( Figure 5C).

Unlike the K48 ubiquitination that mediates protein degradation, K63 ubiquitination mainly mediates changes in cell signaling and affects protein localization. Even so, VSIG2 is not an E3 ubiquitin ligase or deubiquitinating enzyme, and how it changes the ubiquitination level of ANXA2 is still unclear. Previous studies have shown that the E3 ubiquitin ligase FBXW10 mediates the change in the K63 ubiquitination level of ANXA2 [22]. We speculated that VSIG2 interferes with the formation of the FBXW10/ANXA2 complex and affects its modification process. First, we confirmed that FBXW10 can form a complex with ANXA2 and mediate K63 ubiquitination of ANXA2 in gastric cancer ( Figure 5D,E). Further detection revealed that VSIG2-mediated K63 ubiquitination of ANXA2 was dependent on FBXW10 ( Figure 5F). However, the expression level of FBXW10 was not altered by VSIG2 ( Figure 5G). We subsequently explored the relationships among VSIG2, FBXW10 and ANXA2. Co-IP results revealed that when VSIG2 was overexpressed, the binding of FBXW10 to ANXA2 was significantly reduced. In contrast, when *VSIG2* was silenced, the binding of ANXA2 to FBXW10 was significantly increased ( Figure 5H). In addition, we transfected different doses of VSIG2 plasmids into HGC27 cells. Co-IP detection revealed that the binding of FBXW10 to ANXA2 gradually decreased with increasing VSIG2 plasmid concentration ( Figure 5I). This finding is consistent with our hypothesis that VSIG2 competitively binds to ANXA2 with FBXW10, reduces K63 ubiquitination of ANXA2 by FBXW10, and then mediates the membrane expression of ANXA2.

### VSIG2 suppresses GC progression via the ANXA2/NF-κB pathway

**Figure 6:**
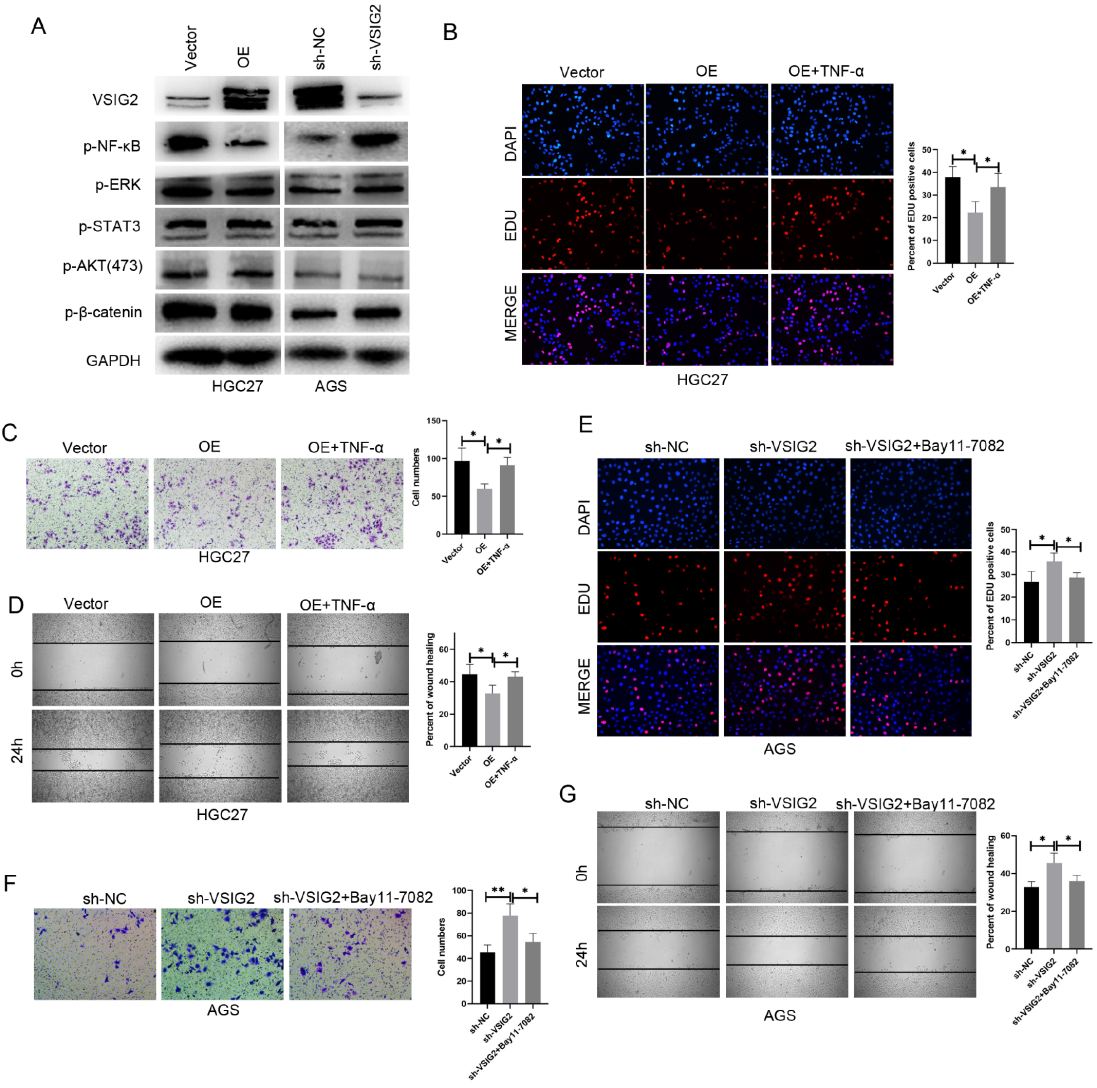
Figure 6 VSIG2 suppresses GC progression via the ANXA2/NF-κB pathway (A) Classical pathways were detected in GC cells with VSIG2 knockdown or overexpression. HGC27 cells with VSIG2 overexpression were treated with the NF-κB agonist TNF-α. (B–D) Then EdU, Transwell and wound healing assays were performed to determine the role of NF-κB in VSIG2-mediated GC progression. AGS cells with VSIG2 knockdown were treated with the NF-κB inhibitor Bay11-7082. (E–G) Then EdU, Transwell and wound healing assays were performed to determine the role of NF-κB in VSIG2-mediated GC progression. *P < 0.05, **P < 0.01.

Previous studies have shown that ANXA2 can mediate the activation of the NF-κB, PI3K-AKT, JAK-STAT3, ERK, β-catenin and other pathways [ 22– 26]. We wanted to determine whether VSIG2-mediated changes in ANXA2 cell localization affect the transduction of signaling pathways. Through western blot analysis, we found that the NF-κB signaling pathway was significantly altered ( Figure 6A). To determine the role of the NF-κB signaling pathway in VSIG2-mediated GC progression, we stimulated VSIG2-overexpressing HGC27 cells with the agonist TNF-α. The EdU results revealed that activation of the NF-κB signaling pathway significantly restored the DNA synthesis ability inhibited by VSIG2 ( Figure 6B). The results of the transwell and wound healing experiments were the same ( Figure 6C,D). To further verify the key role of the NF-κB signaling pathway, we treated *VSIG2*-knockdown AGS cells with the inhibitor Bay11-7082. The results of the EdU, Transwell and scratch assays revealed that inhibition of the NF-κB pathway significantly reversed *VSIG2* knockdown-mediated cell proliferation and migration ( Figure 6E–G). The above experimental results show that the ability of VSIG2 to inhibit the progression of gastric cancer depends on the ANXA2/NF-κB pathway.

## Discussion

GC is a highly heterogeneous malignancy, and its progression is influenced by various factors, including the activation of intrinsic oncogenes and alterations in the external microenvironment [ 27, 28]. This study positions VSIG2 as a previously unrecognized gatekeeper of GC and identifies the VSIG2-ANXA2-NF-κB axis as a critical signaling node. By integrating functional tumor assays and mechanistic analysis, our study is the first to report that VSIG2 acts as a membrane-anchored negative regulator of ANXA2 trafficking rather than a classical immune checkpoint. The co-IP/IF data demonstrated that VSIG2 and ANXA2 co-localise exclusively at the cytoplasmic face of the plasma membrane. VSIG2 overexpression sequesters ANXA2 in the cytosol, whereas *VSIG2* knockdown promotes ANXA2 nuclear accumulation and subsequent NF-κB activation. This spatial confinement is reminiscent of the mechanism described for CD82-mediated retention of EGFR at the cell surface [29], suggesting that VSIG2 may function as a “molecular brake” that limits the oncogenic signaling pool of ANXA2.

ANXA2 is a Ca²-regulated phospholipid-binding protein that shuttles between the cytoplasm, plasma membrane and nucleus to co-ordinate membrane trafficking, actin remodelling and mRNA transport [30]. Emerging evidence indicates that ANXA2 activity and localization are critically governed by ubiquitin signalling, yet the rules that dictate when, where and how ANXA2 is ubiquitinated remain fragmentary. Early work mapped multiple lysines (K28, K47, K81, K115, K148, K204, K227, K279, K302, K313, K324, and K329) as potential acceptor sites for ubiquitin attachment. Site-directed mutagenesis coupled with LC-MS/MS revealed that K28 is the principal residue for K63-linked poly-ubiquitin assembly; this modification is installed by the E3 ligase FBXW10 in response to S6K1-mediated phosphorylation and is counter-balanced by the deubiquitinase USP4. Importantly, K63 ubiquitylation does not destabilize ANXA2; instead, it allows ANXA2 to interact with the p50/p65 subunits of NF-κB, thereby amplifying inflammatory transcription programmes in gastric and oesophageal carcinomas [ 31, 32]. Conversely, K48/K11-linked ubiquitin chains assembled by FBXW7 or TRIM65 target ANXA2 for 26S proteasomal degradation, leading to protein loss in FBXW7-deficient esophageal squamous cell carcinoma and conferring chemoresistance by upregulating MAPK signaling [25]. The regulatory cross-talk between ubiquitination and acetylation further fine-tunes ANXA2 fate: the acetyltransferase p300 acetylates K10 of ANXA2, which enhances USP4 recruitment, decreases K63 ubiquitin density and dampens NF-κB output [33]. Thus, ANXA2 exists as “ubiquitin switchboard” whose polyubiquitin topology (K63 vs K48/K11) and acetylation status jointly determine whether the protein functions as a signaling scaffold or is destined for destruction [34]. Decoding this combinatorial ubiquitin code will be essential for designing isoform-specific ANXA2-directed therapies. Our study is the first to report that VSIG2 competes with FBXW10 for binding to ANXA2 and relies on FBXW10-mediated K63 polyubiquitination of ANXA2 to induce membrane localization of ANXA2 and further inactivate NF-κB, thereby suppressing GC progression.

Even so, there remain limitations in our study. First, the mechanistic studies were performed primarily in adherent GC cell lines; validation in patient-derived organoids and syngeneic mouse models is warranted. Second, the upstream signals that repress VSIG2 transcription in GCs remain unknown. Chromatin-accessibility assays (ATAC-seq) revealed a putative NF-κB binding motif within the VSIG2 promoter, suggesting the possibility of a negative feedback loop. Finally, the role of VSIG2 in other gastrointestinal malignancies and its interaction with immune checkpoint inhibitors merit systematic exploration.

In summary, our work establishes VSIG2 as a membrane-localized tumor suppressor that restrains GC progression by blocking FBXW10-mediated K63 ubiquitination of ANXA2 and subsequent NF-κB activation. Restoring VSIG2 expression or pharmacologically disrupting the ANXA2–FBXW10 axis might represent a promising therapeutic avenue for high-risk gastric cancer patients.

## Supporting information

25753Supplementary_Table_S1

## Supplementary Data

Supplementary data is available at *Acta Biochimica et Biophysica Sinica* online.
