# Supplementary material for: VSIG2 hinders gastric cancer progression by suppressing ANXA2-mediated NF-κB pathway activation: VSIG2 hinders gastric cancer progression
Source: Acta Biochim Biophys Sin (Shanghai). 2025 Nov 4;57(11):1834–46. doi: 10.3724/abbs.2025202 (PMC12666673; doi:10.3724/abbs.2025202)
Supplement: 25753Supplementary_Table_S1 [file 25753Supplementary_Table_S1.docx]

| **Supplementary Table S1. Sequences of primers used in this study** | |
| --- | --- |
| Gene | Sequence (5′→3′) |
| *VSIG2* F: CATCTCTGAGTCCCATCCAATCC  R: TGACCCGCTTTGACTTAGAAC  *ANXA2* F: TCTACTGTTCACGAAATCCTGTG  R: AGTATAGGCTTTGACAGACCCAT  *GAPDH* F: ACAACTTTGGTATCGTGGAAGG  R: GCCATCACGCCACAGTTTC | |
